# Homology Directed Knockin of Point Mutations in the Zebrafish *tardbp* and *fus* Genes in ALS Using the CRISPR/Cas9 System

**DOI:** 10.1371/journal.pone.0150188

**Published:** 2016-03-01

**Authors:** Gary Alan Barclay Armstrong, Meijiang Liao, Zhipeng You, Alexandra Lissouba, Brian Edwin Chen, Pierre Drapeau

**Affiliations:** 1 Department of Neurosciences, Research Centre of the University of Montréal Hospital Centre, Montréal, Québec, Canada; 2 Department of Neurology and Neurosurgery, Research Institute of the McGill University Health Centre and Centre for Research in Neuroscience, Montréal, Québec, Canada; SRI International, UNITED STATES

## Abstract

The methodology for site-directed editing of single nucleotides in the vertebrate genome is of considerable interest for research in biology and medicine. The clustered regularly interspaced short palindromic repeats (CRISPR)/CRISPR-associated protein 9 type II (Cas9) system has emerged as a simple and inexpensive tool for editing genomic loci of interest in a variety of animal models. In zebrafish, error-prone non-homologous end joining (NHEJ) has been used as a simple method to disrupt gene function. We sought to develop a method to easily create site-specific SNPs in the zebrafish genome. Here, we report simple methodologies for using CRISPR/Cas9-mediated homology directed repair using single-stranded oligodeoxynucleotide donor templates (ssODN) for site-directed single nucleotide editing, for the first time in two disease-related genes, *tardbp* and *fus*.

## Introduction

In humans, mutations in *TARDBP* (coding for TDP-43, a major protein component of inclusions in many neurodegenerative diseases) or *FUS* cause the progressive disease amyotrophic lateral sclerosis (ALS) and in rare cases frontotemporal lobar degeneration (FTLD) [1–5]. The majority of these mutations are missense mutations caused by base pair substitutions clustered in regions highly conserved across vertebrates. Many transgenic models have been generated but knockin of point mutations has not been reported, yet would provide the most disease-relevant models. ALS-associated mutations in *TARDBP* are commonly found in the C-terminal glycine-rich region and in the C-terminal of *FUS* encoding a nuclear localization signal [5]. We have previously demonstrated that expression of mutant human *TARDBP*^A382T^ or *FUS*^R521H^ mRNA causes motor deficits and aberrant motor axon projections in larval zebrafish [6,7]. However, these transient human mRNA expression models as well as other loss-of-function (early lethality) models [8,9] limit investigations to the first few days of zebrafish development and do not address gain-of-function disease phenotypes as may be the case for ALS which manifests clinically in midlife. Ideally, one would like to introduce these specific point mutations in the genes of interest and follow the resulting phenotypes over a long time course.

**Fig 1:**
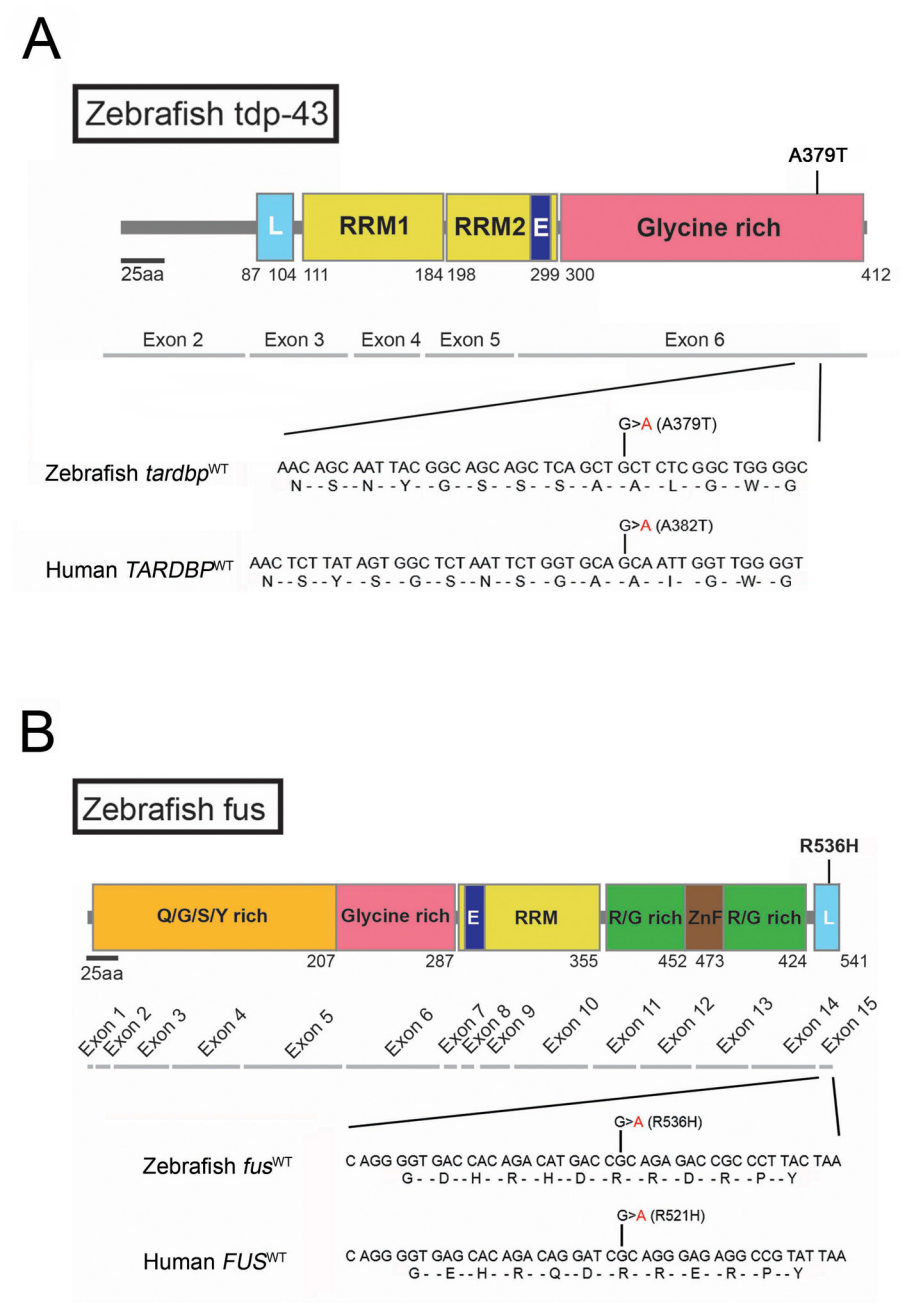
HDR knockin of point mutations in zebrafish using the CRISPR/Cas9 system with ssODN templates. Schematic representation of zebrafish tdp-43 (**A**) and fus (**B**) and locations of point mutations encoding missense mutations generated by HDR (top). L, nuclear localization sequences; E, nuclear export sequences; RRM, RNA recognition motifs; ZnF, zinc finger motif. Exon coding sequences (middle). Comparisons of zebrafish gRNA target sites with human coding sequences (bottom). Note the high amino acid sequence homologies between human and zebrafish proteins. ALS-causing point mutations (red) encoding missense mutations *TARDBP*^A382T^ and *FUS*^R521H^ are indicated in the human sequences and analogous point mutations are noted in the zebrafish genes (*tardbp*^A379T^ and *fus*^R536H^).

Editing of the zebrafish genome, using TALEN- or CRISPR/Cas9-based homology-dependent repair (HDR), has been previously used to incorporate sequences (though not point mutations) encoding a restriction site or a modified *loxP* site in two different loci [10] or and convert eGFP into Gal4 transgenic lines [11]. A similar method using the CRISPR/Cas9 system has been used in *C*. *elegans* [12], *Drosophila* [13] and mice [14] to introduce defined point mutations, but as of yet not in zebrafish. We sought to develop a general CRISPR/Cas9 methodology in zebrafish that permits the generation of knockin lines, in our case with the corresponding disease-causing point mutations in humans, zebrafish *tardbp*^A379T^ (*TARDBP*^A382T^) and *fus*^R536H^ (*FUS*^R521H^), as these are among the first mutations to be identified in patients with ALS [1,3,4]. Moreover, gene and amino acid sequence homology at these loci are highly conserved between humans and zebrafish (Fig 1A and 1B).

## Materials and Methods

### Zebrafish husbandry and ethical considerations

Wild type TL adult zebrafish (*Danio rerio*) were maintained at 25°C under a 12/12 hour light/dark cycle in a colony at the animal research facility at the Centre Hospitalier de l’Université de Montréal Research Centre (CRCHUM) located in Montréal Québec, Canada. Embryos and larvae were raised at 28.5°C under at 12/12 hour light/dark cycle. Genomes of entire 48 hour old embryos were extracted in order to measure genomic indels. The Animal Care Committee of the University of Montreal Research Centre approved all experiments.

### Synthesis of Cas9 mRNA and gRNAs

Synthesis of zebrafish-optimized nls-zCas9-nls mRNA was done using previously described methods [15] and the pCS2-nCas9n was a gift from Wenbiao Chen (Addgene plasmid # 47929). Briefly, the nls-zCas9-nls template was linearized with NotI and synthesized using the mMESSAGE mMACHINE SP6 kit (Ambion/Invitrogen) followed by a phenol-chloroform extraction and ethanol precipitation. The pT7-gRNA was a gift from Wenbiao Chen (Addgene plasmid # 46759). The pT7-gRNA was linearized with BamHI and RNA was transcribed using the MEGAshortscript T7 kit (Ambion/Invitrogen) and extracted and precipitated using the same methods for nls-zCas9-nls mRNA.

The sequences of the designed gRNAs are as follows:

*tardbp*: GGCAGCAGCTCAGCTGCTCT (forward strand, targeting exon 6);

*fus*: TAGTAAGGGCGGTCTCTG (reverse strand, targeting exon 15).

### Donor templates

Single-stranded oligodeoxynucleotide donor templates (ssODN) were designed with the following sequences and synthesized by Invitrogen:

*tardbp* template: GGCAGCAGCTCAGCTACTCTCGG

*fus* template: CCACAGACATGACCACAGAGACCGCCCTTACTA

*tardbp* template with silent mutations: CCAAACATATAGCTCGGCTAACAGCAATTACG GCAGCAGCTCAGCCACTTTGGGTTGGGGCACCGGCTCTAACTCGGGCGCTGCCAGTGCTGGCTTTAAC

### Micro-injection of embryos

A solution containing either the Cas9 mRNA (200 ng/μl) and gRNA (30 ng/μl) or Cas9 (200 ng/μl) and gRNA (30 ng/μl) and ssODN (60 ng/μl) were injected into one-cell stage zebrafish eggs. A total volume of 1.5 nl was injected into each embryo.

### Restriction fragment length polymorphism assay

Genomic DNA was extracted from individual 48 hpf larvae using the REDExtract-N-AMP Tissue PCR kit (Sigma-Aldrich) and used as a template for PCR using the following primer sets:

*tardbp*-forward: CAGTACGGAGAGGTCACAGACG

*tardbp*-reverse: CACGCTAGGAATACCGACAC

*fus*-forward: CCTGGAAAGATGGACTCGAGGTG

*fus*-reverse: TAGTACTAAGGTGGCCTAACCGC

Amplified PCR products of 752 and 409 base pairs for *tardbp* and *fus* were digested with PvuII or MwoI for *tardbp* and *fus* amplicons respectively. Digested PCR amplicons generated bands of 609 and 143 base pairs for *tardbp* and 274 and 135 for *fus*. Indels were identified based upon the loss of restriction sites and confirmed by sequencing of homozygous amplicons using an Applied Biosystems 3730xl DNA Analyzer (Thermo Fisher Scientific).

## Results

### CRISPR/Cas9-mediated NHEJ failed to generate single nucleotide substitutions

**Fig 2:**
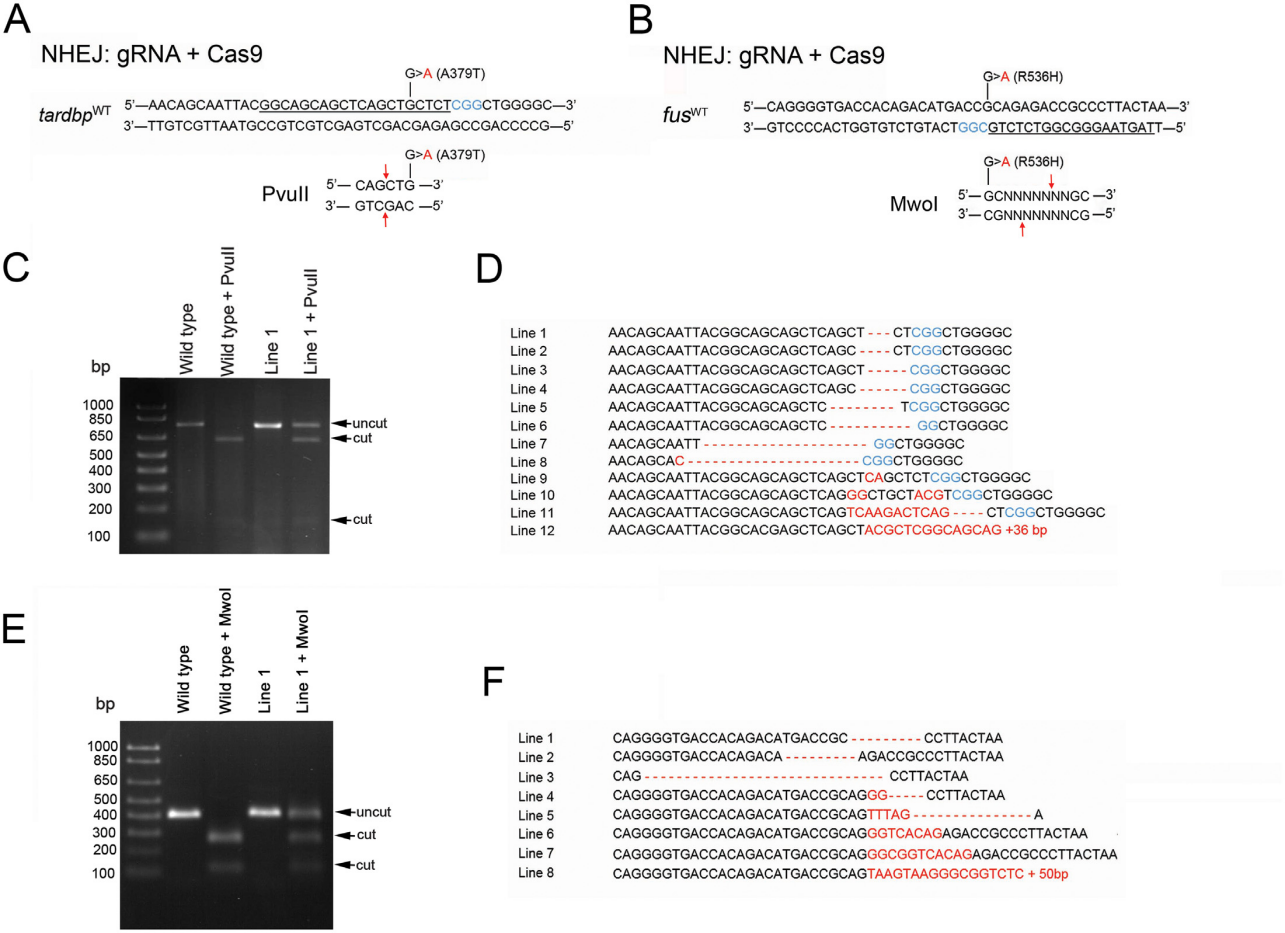
NHEJ reliably generated indels at target site but failed to generate point mutations. Design of gRNAs targeting our regions of interest in the zebrafish *tardbp* (**A**) and *fus* (**B**) were generated and co-injected with Cas9 into the one cell stage fertilized egg. gRNA target sites are underlined and PAM sequences are denoted in blue. Germline transmission of mutations was assessed by RFLP analysis. PCR amplicons for *tardbp* and *fus* were designed to contain unique restriction enzyme sites (PvuII and MwoI for *tardbp* and *fus* respectively) that would fail to cut *tardbp*^A379T^ and *fus*^R536H^ mutant amplicons. **C**, Example gel electrophoresis of PCR amplicons of wild type and an example heterozygotes mutant *tardbp* line. Note the partial cut in our mutant line digested with PvuII **D**, Sequencing of homozygous F2 larval amplicons for *tardbp*. **E**, Example gel electrophoresis of PCR amplicons of wild type and an example heterozygotes mutant *fus* line. Note the partial cut in our mutant line digested with MwoI **F**, Sequence results of homozygous F2 larval amplicons for our *fus* mutant lines. None of our generated lines were single point mutations suggesting that CRISPR/Cas9-medated NHEJ was not a desirable method for generating specific point mutations.

In an initial series of experiments we explored the feasibility of generating point mutations using CRISPR/Cas9-mediated NHEJ in the zebrafish *tardbp* and *fus* genes. A gRNA targeting the glycine-rich C-terminal portion of the *tardbp* gene and another targeting the nuclear localization sequence of the *fus* gene were selected based upon low sequence homology to other genomic regions and position of the protospacer adjacent motif (PAM) close to the sites of the disease-causing point mutations (Fig 2A and 2B). Microinjection of the gRNA and Cas9 mRNA at the one-cell embryo stage was performed using standard techniques [15]. Adult (F0) zebrafish were crossed with wild type animals and F1 larvae were screened at 48 hours post-fertilization (hpf) for genomic modifications at the target loci. PCR amplicons containing the target loci were analyzed using restriction fragment length polymorphism (RFLP) to determine the presence of mutations. The restriction enzymes, PvuII and MwoI for *tardbp* and *fus*, respectively, were selected based upon their potential for identifying single nucleotide substitutions that corresponded to the desired point mutations (Fig 2C and 2D). Twelve of 58 adult F0 *tardbp*-targeted zebrafish successfully transmitted indels to their F1 progeny representing a 21% efficiency in germline transmission. For the *fus*-targeted F0 adults, 8 of 46 transmitted unique indels (17% efficiency) to their F1 progeny. Individual *tardbp* and *fus* founders displayed differing indel transmission rates (Table 1). Outcrossed F1 zebrafish were raised to adulthood and incrosses were performed and homozygous larval PCR amplicons were sequenced. Seven lines that targeted *tardbp* resulted in deletions, 3 lines had insertions and 2 lines had a combination of insertions and deletions (Fig 2E). Similar indel distributions for our *fus*-targeted lines were observed: 3 lines had deletions, 3 lines had insertions and 2 lines had both insertions and deletions encompassing our target site (Fig 2F). Although CRISPR/Cas9-mediated NHEJ reliably generated indels, none of our derived lines resulted in the generation of single nucleotide substitutions.

**Table 1:** Indel transmission from F0 founders to F1 progeny.

*tardbp*	Fraction	*fus*	Fraction
Line 1	6/12	Line 1	3/11
Line 2	2/7	Line 2	1/7
Line 3	2/13	Line 3	4/14
Line 4	2/14	Line 4	3/7
Line 5	1/14	Line 5	2/7
Line 6	4/7	Line 6	2/7
Line 7	1/7	Line 7	10/14
Line 8	1/7	Line 8	1/14
Line 9	1/7		
Line 10	2/24		
Line 11	1/7		
Line 12	3/7		

### Homology-dependent repair following CRISPR/Cas9 DNA double-stranded cuts introduced the desired single nucleotide substitutions

**Fig 3:**
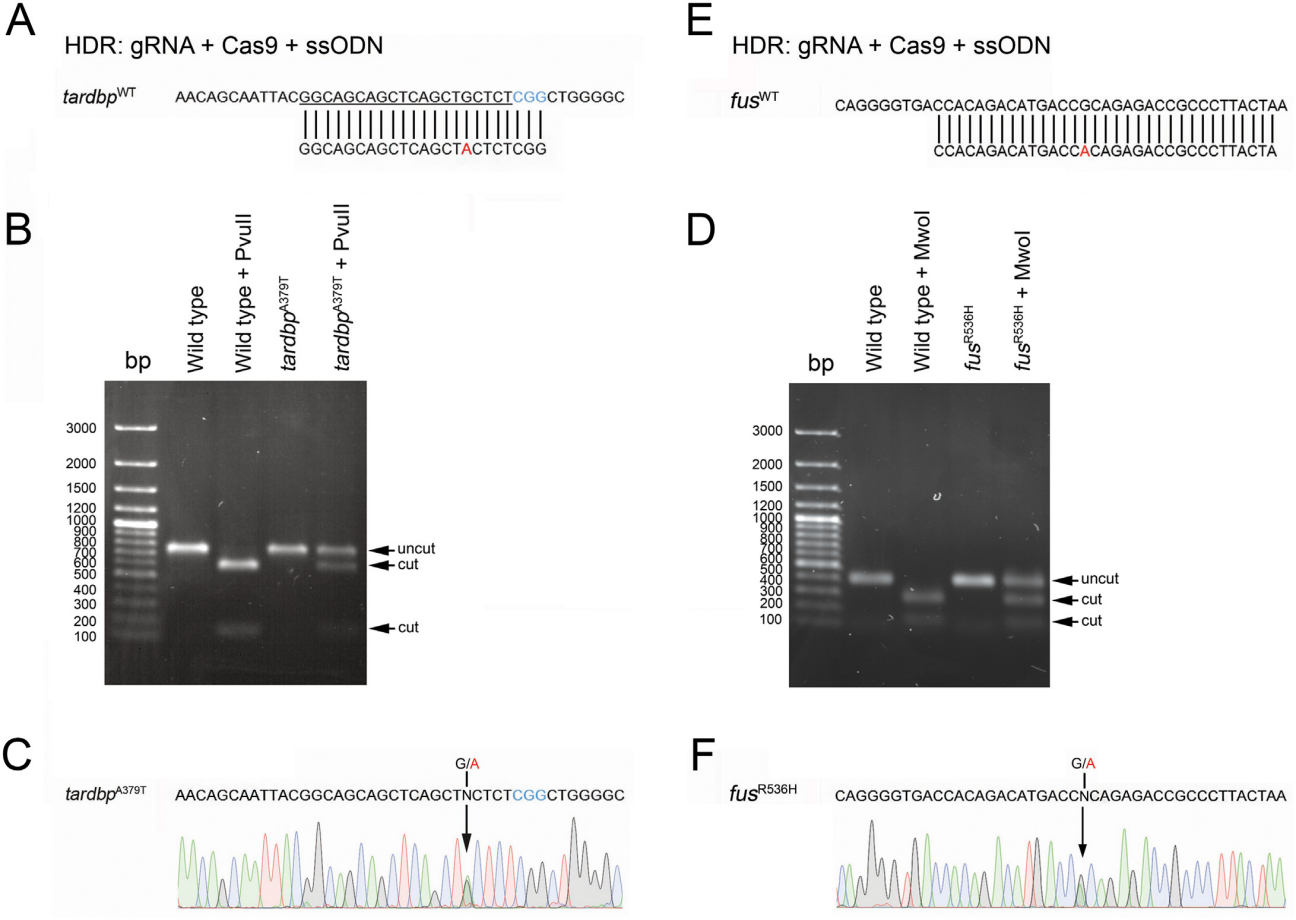
Generation of point mutations encoding tardbp^A379T^ and fus^R536H^ was achieved by HDR. Co-injection of our gRNA, Cas9 and an ssODN template encoding our desired point mutations encoding *tardbp*^A379T^ (**A**) and *fus*^R536H^ (**E**) was made into one cell stage embryos. gRNA target sites are underlined and PAM sequences are denoted in blue. We identified a line in each batch of raised *tardbp* and *fus* F0 fish that transmitted an indel, identified by RFLP (**B** and **D**) that integrated and transmitted to F1 progeny the *tardbp*^A379T^ (**C**) and *fus*^R536H^ (**F**) missense point mutations identified following sequencing. Corresponding electropherograms of heterozygous F1 progeny indicating our desired point mutations (arrowheads).

To address the feasibility of using HDR to introduce point mutations of interest, we designed ssODN template sequences containing point mutations, which were then co-injected with gRNA and Cas9 mRNA. Flanking the G to A point mutation in the ssODN *tardbp* template were 15 base pair homology arms on the 5’ and 7 base pairs on the 3’ sides (Fig 3A). We designed a template that spanned the same region targeted by the gRNA as this is where DNA cleavage was expected to occur. The likelihood that the gRNA would also target the template was of concern. However single nucleotide mismatches near the PAM site in the gRNA have been reported to be less effective at generating double-stranded DNA breaks [16]. We thus speculated that the ssODN template containing the G to A point mutation 5 base pairs from the PAM sequence might not be recognized by the gRNA. A total of 46 adult zebrafish were raised and outcrossed with wild type animals and F1 larvae were screened using RFLP. Fourteen of 46 adults successfully transmitted (30% efficiency) indels to their F1 progeny but only 1 of these adult zebrafish transmitted the proper edited sequence encoding the zebrafish *tardbp*^A379T^ point mutation (Fig 3B and 3C).

We also designed an ssODN template sequence for HDR integration of the *fus*^R536H^ missense mutation. Flanking the G to A point mutation in this ssODN template were 14 and 18 base pair 5’ and 3’ homology arms (Fig 3B). Unlike the *tardbp* ssODN template that spanned the gRNA target site, the point mutation in the *fus* ssODN template was contained in the PAM sequence located at the nucleotide adjacent to the gRNA sequence. A successful integration of our point mutation would not change the PAM sequence from NGG and it was of concern that subsequent DNA cleavage would occur following template integration. Despite this possibility we were able to generate our point mutation of interest. A total of 47 F0 zebrafish were raised and crossed with wild type animals to test for HRD heritable integration of the point mutation encoding the *fus*^R536H^. Twelve of 47 F0 zebrafish transmitted indels to their F1 progeny (26% efficiency) but only 1 of these adult fish successfully transmitted the G to A point mutation to its progeny (Fig 3E and 3F).

**Fig 4:**
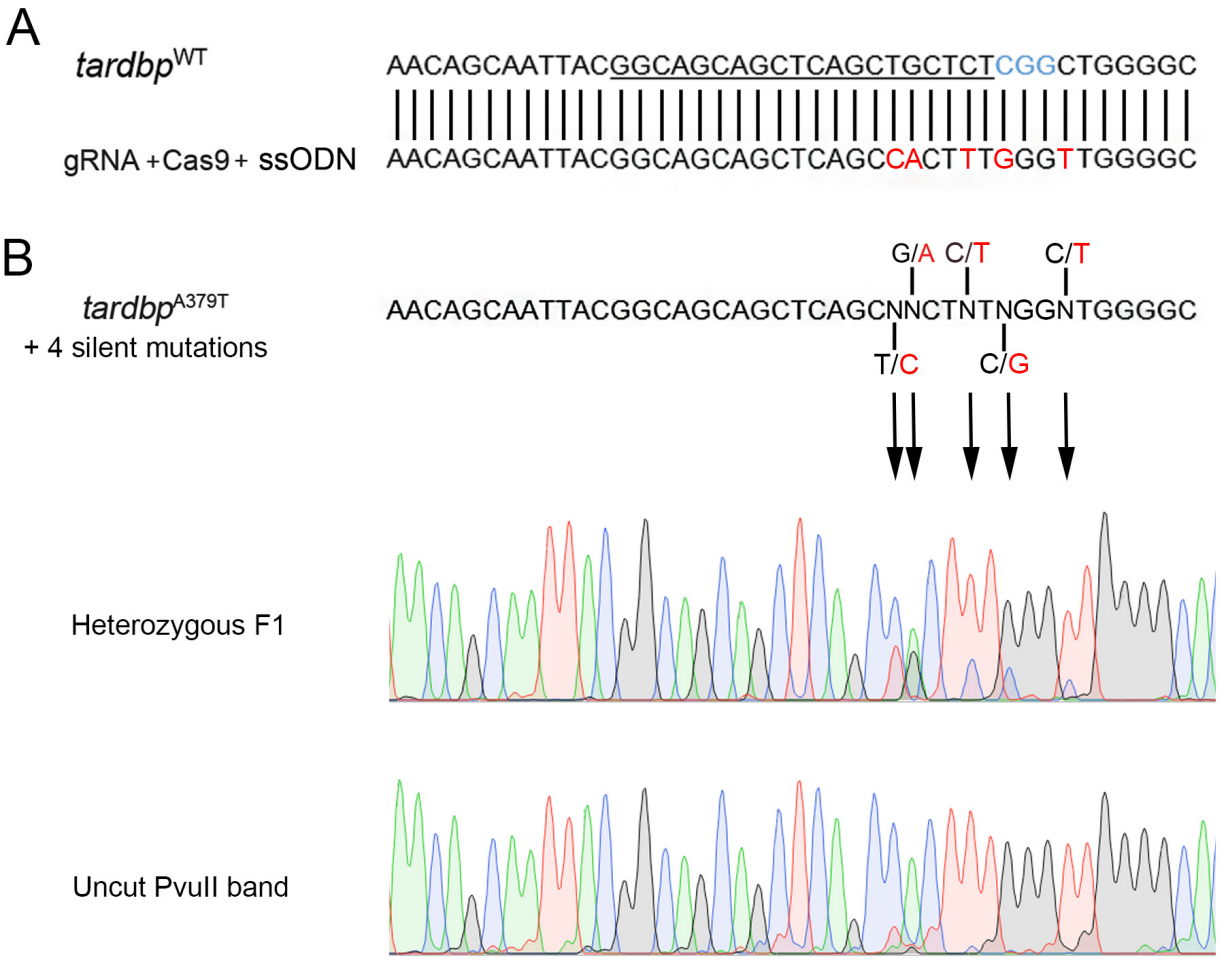
HDR ssODN template integration was confirmed by the inclusion of multiple silent around the tardbp^A379T^ point mutation. Confirmation of ssODN integration was achieved by co-injection of an ssODN template containing our point mutation of interest and 4 silent point mutations (red nucleotides; **A** shown for the region flanking the mutation) and was successfully transmitted to F1 progeny; note the double peaks in the electropherogram of heterozygous F1 progeny (top electropherograms; **B**). We also sequenced the undigested PvuII band and confirmed the integrated template (bottom electropherogram).

Despite the successful generation of our point mutations in the experiments in which we co-injected ssODN templates, this does not rule out the possibility that these mutations were a result of the random repair by NHEJ and not HDR. To investigate if HDR could be responsible for integration of a template we designed a *tardbp* template bearing four silent mutations flanked by 46 and 53 base pair 5’ and 3’ arms from the A379T mutation of interest as this set of mutations could only be generated by HDR and not randomly e.g. by NHEJ (Fig 4A). A total of 77 F0 zebrafish were raised and crossed with wild type animals to test for HRD heritable integration of the template. We were able to detect this integration in 3 heterozygous F1 larvae from separate F0 founders (Fig 4B), confirming editing by HDR using this template. This represents a 4% efficiency for heritable transmission of the template containing 5 mutations.

## Discussion

Taking advantage of the HDR pathway we were able to generate defined missense mutations in the zebrafish *tardbp* and *fus* genes that are analogous to those in their human orthologs associated with ALS. We also demonstrated that donor templates containing defined point mutations, within the gRNA target site, can be utilized to knockin point mutations despite the increased likelihood that the gRNA would recognize the ssODN template sequences. This may account for the low efficiencies for heritable integration of defined point mutations. However, similar experiments in mice suggest that donor templates that are closer to the gRNA target site more frequently incorporate defined point mutations when compared to gRNAs targeting sequences further from the target site [14]. We also examined two templates, one 23 and the other 100 base pairs in length for HDR-mediated integration at the *tardbp* gRNA target site. Although a single F0 founder (1/46) was identified for the smaller template *vs*. 3/77 F0 founders for the longer template, further investigations pertaining to the optimization of template integration with respect to template length and location to the PAM sequence would be useful. Furthermore, the efficiency of HDR would also be limited by the overall efficiency of Cas9-mediated cleavage at the gRNA target site. Here we report efficiencies of 21% and 17% Cas9-mediated cleavage for our *tardbp* and *fus* gRNA target sites and we speculate that if these efficiencies were higher better integration of our template would have occurred.

NHEJ repairs double-stranded DNA breaks often with indels at the cleaved DNA site. CRISPR/Cas9-mediated NHEJ can be used to disrupt gene function either by targeted removal of start codons, frameshifts, or the generation of premature stop codons, effectively generating loss-of-function knockout models. These are useful for exploring the fundamental aspects of altered cellular biology but are of limited value for modeling phenotypes not caused by loss-of-function. The ability to generate point mutations at predefined loci by HDR using the CRISPR/Cas9 system has several advantages. First, traditional transgenesis relies upon the knockin of cDNA, often under the control of a non-endogenous promoter. Differing levels of protein expression and variations in the promoter expression patterns can impact the phenotype and possibly lead to erroneous results. Furthermore, expression of the endogenous gene usually continues in the presence of the transgenic allele. Some proteins such as TDP-43 and FUS autoregulate their expression, necessitating careful measurements of transgenic and endogenous protein levels. Moreover, limited conclusions can be drawn from experiments where a human gene, with reduced homology to the ortholog, is expressed in a phylogenetically removed species. The CRISPR/Cas9-mediated cleavage and HDR ssODN template knockin avoids these limitations by editing the endogenous gene. Second, unlike the labour and time intensive mouse ES cell-derived mutant lines, in zebrafish genomic insertion efficiencies can be determined within two days and stable transmission can be determined when sexually mature adults are outcrossed (2–3 months).

In conclusion, we have established a simple experimental methodology for introducing defined point mutations in the zebrafish genome by editing specific loci. We demonstrated that NHEJ using gRNAs targeting either *tardbp* or *fus* failed to generate single nucleotide substitutions at our desired targets despite having gRNA target sites near the nucleotide of interest. However, co-injection of an ssODN template was sufficient to knockin the desired point mutation encoding two missense mutations (*tardbp*^A379T^ and *fus*^R536H^) relevant to ALS and for the first time in models of these genetic disorders. These lines need however to be out-crossed over several generations to eliminate any potential off-target mutations. We believe that this technique will be useful for the creation of other disease models and in general for editing point mutations in the zebrafish genome.
